# What Is the Appropriate Sample Size in Human Cadaveric Studies? A Quantitative Review of 770 Articles

**DOI:** 10.1002/ca.70007

**Published:** 2025-07-21

**Authors:** Joe Iwanaga, Kyoichi Obata, Tomotaka Kato, Rarinthorn Samrid, Emma R. Lesser, Juan J. Cardona, Keishiro Kikuchi, Chung Yoh Kim, Kisho Ono, Anthony D'Antoni, Noritaka Komune, Yoko Tabira, Mi‐Sun Hur, Norio Kitagawa, Hee‐Jin Kim, Marios Loukas, Koichi Watanabe, R. Shane Tubbs

**Affiliations:** ^1^ Department of Neurosurgery Clinical Neuroscience Research Center, Tulane University School of Medicine New Orleans Louisiana USA; ^2^ Department of Neurology, Clinical Neuroscience Research Center Tulane University School of Medicine New Orleans LA USA; ^3^ Department of Structural & Cellular Biology Tulane University School of Medicine New Orleans Louisiana USA; ^4^ Department of Neurosurgery and Ochsner Neuroscience Institute Ochsner Health System New Orleans Louisiana USA; ^5^ Dental and Oral Medical Center Kurume University School of Medicine Kurume, Fukuoka Japan; ^6^ Division of Gross and Clinical Anatomy, Department of Anatomy Kurume University School of Medicine Kurume, Fukuoka Japan; ^7^ Department of Oral and Maxillofacial Surgery Graduate School of Medicine, Dentistry and Pharmaceutical Sciences, Okayama University Okayama Japan; ^8^ Division of General Dentistry Nippon Dental University Hospital Chiyoda‐ku, Tokyo Japan; ^9^ Department of Anatomy, Faculty of Medicine Khon Kaen University Khon Kaen Thailand; ^10^ Department of Orthopaedic Surgery Kurume University School of Medicine Fukuoka Japan; ^11^ Department of Anatomy Dongguk University School of Medicine Gyeongju South Korea; ^12^ Department of Medical Education and Scholarship Rowan‐Virtua School of Osteopathic Medicine Stratford NJ USA; ^13^ Department of Otorhinolaryngology Graduate School of Medical Sciences, Kyushu University Fukuoka Japan; ^14^ Department of Anatomy Daegu Catholic University School of Medicine Daegu South Korea; ^15^ Department of Oral and Maxillofacial Anatomy Graduate School of Medical and Dental Sciences, Institute of Science Tokyo Tokyo Japan; ^16^ Division in Anatomy and Developmental Biology, Department of Oral Biology, Human Identification Research Institute, BK21 FOUR Project Yonsei University College of Dentistry Seoul South Korea; ^17^ Department of Anatomical Sciences St. George's University, School of Medicine Grenada West Indies; ^18^ Department of Pathology St. George's University, School of Medicine Grenada West Indies; ^19^ Nicolaus Copernicus Superior School College of Medical Sciences Olsztyn Poland; ^20^ Department of Clinical Anatomy Mayo Clinic Rochester Minnesota USA; ^21^ Department of Surgery Tulane University School of Medicine New Orleans Louisiana USA; ^22^ University of Queensland Brisbane Australia

**Keywords:** anatomy, cadaveric study, evidence, evidence‐based, number, sample size, specimens

## Abstract

Determining an appropriate sample size in human cadaveric studies remains a long‐standing and unresolved challenge. Unlike other basic science fields, anatomical research is constrained by factors such as limited human donor availability, cultural considerations, and ethical restrictions. Despite these limitations, researchers are often asked to justify sample sizes, yet no standardized guidelines currently exist. To quantitatively assess sample sizes in recent human cadaveric studies and propose evidence‐based recommendations for future research, a PubMed search was conducted on February 26, 2024, using the term *human cadaveric study*. The articles published in 2023 and 2024 were screened, yielding 770 eligible studies. Data extracted included the total sample size, number of classified groups, and journal impact factor (IF). Descriptive statistics, linear regression, and correlation analyses were performed. Continuous variables were summarized using medians and interquartile ranges (IQR). The median sample size was 11.5 (IQR: 7–20), and 47.9% of studies used 10 or fewer specimens. The median number of classified groups was 3 (IQR: 2–4). Linear regression showed that studies dividing specimens into 2–6 groups often failed to meet the recommended sample size per group based on regression modeling. No significant correlation was found between sample size and journal IF (*r* = −0.062, *p* = 0.115). Most cadaveric studies rely on small sample sizes due to inherent constraints, yet many still attempt a subgroup analysis without sufficient statistical power. Although flexibility is essential in anatomical research, we recommend a minimum total sample size of 10 for basic studies and at least five samples per group for those involving classification. Cadaveric sample size alone does not predict journal impact, highlighting the importance of methodological rigor over quantity.

## 1 Introduction

Anatomical studies are inherently subjective. Anatomical research is more susceptible to biases and misinterpretations than other basic science disciplines. These may arise from factors beyond experimental control, such as the dissection approach, religious or cultural considerations, ethnicity, language, and perceptions of death (Iwanaga, Takeda, et al. 2025). Anatomical researchers frequently face a complex but common situation: “There is no way to increase the number of cadavers in this study,” even when prompted by reviewers to do so.

In contrast, animal experiments and clinical studies generally allow for the expansion of sample size unless the study involves rare diseases or highly specialized procedures. Nonetheless, colleagues in other fields often ask anatomists, “What is the ideal sample size for a cadaveric study?” This question remains largely unanswered.

In the broader scientific literature, numerous studies have addressed the question of optimal sample size (Malterud et al. 2016; Vasileiou et al. 2018; Mutedzi et al. 2023), often utilizing power analysis to guide estimation (Meurs 2016). Naturally, the appropriate number of samples depends on the research objective. To our knowledge, the largest sample size in a postmortem context is 57,903, though this was an autopsy‐based study rather than one involving donors, which significantly affects the feasibility (Launiainen and Ojanperä 2014).

In forensic science, several studies have explored the use of postmortem tissues. For example, Hammer et al. (2023) investigated the minimum sample size needed to achieve stable estimation of material properties using human dura mater and scalp, representing two distinct soft tissues. These studies provide some guidance, but do not directly address anatomical research as a whole.

In a world without donor limitations or usage restrictions, the ideal sample size for anatomical studies could follow principles established in noncadaveric research. However, reality imposes significant constraints. Some researchers have attempted to address sample size questions for specific anatomical structures—for instance, in muscle architecture studies using small cohorts (Tuttle et al. 2012). Evidence‐based anatomy (EBA) ushered in the use of anatomical meta‐analyses (AMAs), which have helped overcome the sample size hurdle (Tomaszewski et al. 2018). The idea of pooling data from multiple cadaveric studies in a meta‐analysis can help readers make sense of multiple studies with small sample sizes (D'Antoni et al. 2025). However, oftentimes these studies are heterogeneous, which makes pooling their data problematic.

Still, the fundamental question remains unresolved: What is the ideal sample size for human cadaveric studies? The anatomical community has largely overlooked this long‐standing issue. However, it is time to move beyond the routine justification, “the sample size is small because this is a cadaveric study.” Instead, we should aim to establish baseline reference values—derived from past anatomical studies—that researchers can consult. These values should serve as general guidance, not rigid standards, to preserve the exploratory and descriptive nature of the anatomical sciences.

Therefore, this study aimed to determine a recommended sample size for human cadaveric research by systematically analyzing previously published cadaveric studies while considering the unique circumstances inherent to anatomical research.

## 2 Materials and Methods

A literature search was conducted by the first author on PubMed on February 26, 2024, using the term *human cadaveric study*, yielding over 20,000 results. Only articles published in 2023 and 2024 were included to analyze recent trends, resulting in 1213 records. Titles and abstracts were screened to exclude nonhuman or noncadaveric studies. Only studies using human cadaveric tissue were retained for analysis. Studies involving both cadavers and patients, or those categorized as letters, were excluded. After applying exclusion criteria and full‐text review, eligible articles were finalized for analysis.

A full‐text review was conducted to extract the following data: article title, journal name, total number of cadaveric samples, and number of groups (categories or classifications) defined by the authors. The highest reported group count was used for analysis in studies with multiple group classifications.

Journal impact factor (IF) data were obtained from the 2024 Journal Citation Reports. Due to limited public access during data collection, the open‐source data were compiled from the JCR‐Impact‐Factor‐2024 PDF (ResearchGate 2025).

Continuous variables were summarized using medians and interquartile ranges (IQR). Linear regression assessed the relationship between total sample size and number of groups. Correlation analysis examined associations between sample size or group number and journal IF. A *p* value < 0.05 was considered statistically significant.

All authors confirm adherence to local and international ethical standards for the use of human cadaveric specimens and related imagery in anatomical research (Iwanaga et al. 2021, 2022; Iwanaga, Kim, et al. 2025).

## 3 Results

Of the 1213 articles identified, 770 met the inclusion criteria and were included in the final analysis.

### 3.1 Trends in Sample Sizes of Cadaveric Studies

The median sample size was 11.5 (IQR: 7–20). The mean sample size was 22.8. Of 770 articles, 369 (47.9%) used 10 samples or fewer (Table 1). Of 369 articles, 49 (6.4%) used one sample (e.g., single case report, technical note, introducing cadaveric model) (Table 2).

**TABLE 1:** Number of articles in each sample size group.

Sample size (range)	Articles
1–10	369 (47.9%)
11–20	221 (28.7%)
21–30	65 (8.4%)
31–40	40 (5.2%)
41–50	19 (2.5%)
51–100	35 (4.5%)
101 to	21 (2.7%)
Total	770 (100%)

**TABLE 2:** Number of articles with 10 or fewer samples.

Sample size	Articles
1	49 (6.4%)
2	21 (2.7%)
3	16 (2.1%)
4	15 (1.9%)
5	34 (4.4%)
6	32 (4.2%)
7	31 (4.0%)
8	71 (9.2%)
9	16 (2.1%)
10	84 (10.9%)
Total	369 (47.9%)

### 3.2 Relationship Between Total Sample Size and Number of Groups

**FIGURE 1:**
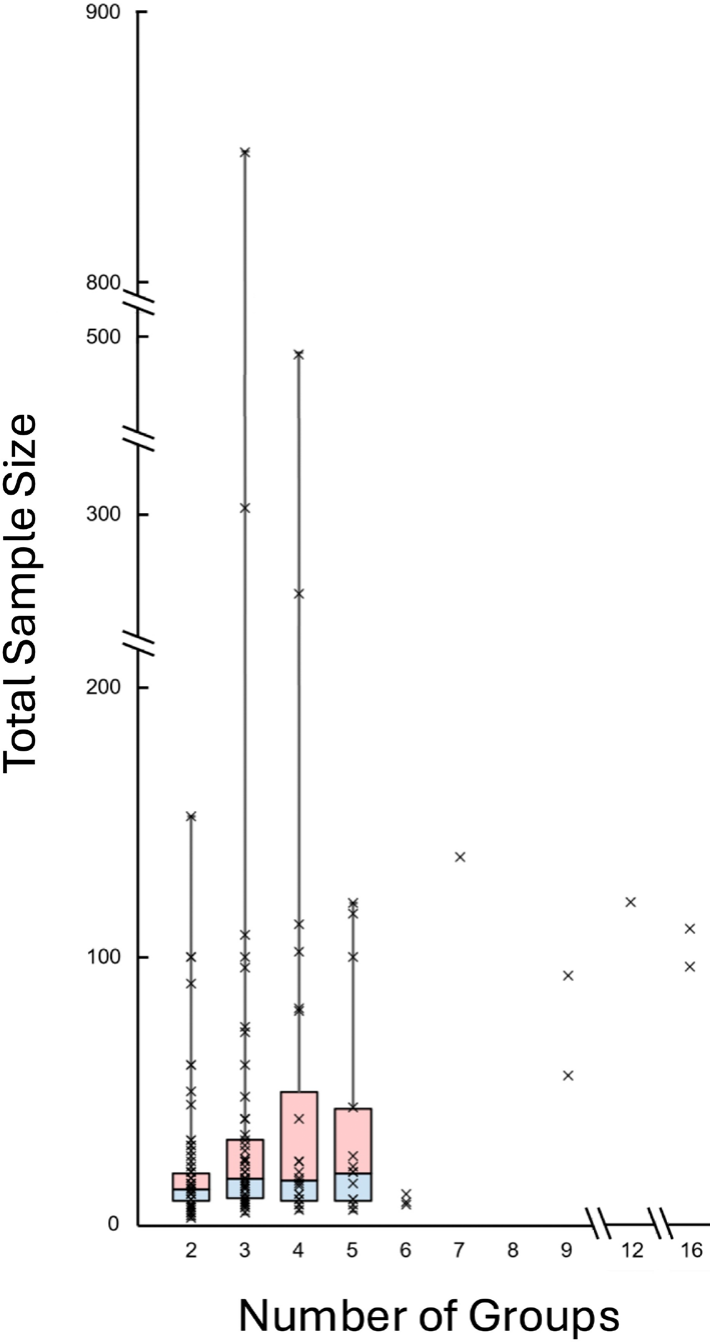
Relationship between total sample size and number of groups.

The median number of groups was 3 (IQR: 2–4) (Figure 1).

A linear regression analysis using the number of groups as the dependent variable and the total sample size as the independent variable yielded the equation:

$$\text{Number of groups}=0.004\times \text{Total sample size}+3.0226\;\left(p=0.022\right)$$

Conversely, when using the total sample size as the dependent variable and the number of groups as the independent variable, the resulting equation was:

Total sample size=7.101×Number of groups+15.690

Based on these equations, the recommended number of samples per group was calculated. Studies with two to six groups, each including four or more samples, did not meet the recommended total sample size (Tables 3 and 4).

**TABLE 3:** Actual and recommended sample sizes calculated using the regression equation.

Number of groups	2	3	4	5	6
Median of actual sample size	14	18	17.5	20	9
Recommended sample size	29.892	36.993	44.094	51.195	58.296

**TABLE 4:** Regression analysis.

	Regression coefficient	Lower 95%	Upper 95%	Standard error	*t*	*p*
Intercept	3.023	2.708	3.352	0.163	18.563	< 0.001
Number of groups	0.004	0.001	0.008	0.002	2.291	0.023

	Regression coefficient	Lower 95%	Upper 95%	Standard error	*t*	*p*
Intercept	15.690	−7.248	38.629	11.622	1.350	0.179
Total sample size	7.101	0.983	13.219	3.100	2.291	0.023

### 3.3 Correlation Between Sample Size and IF

The median IF of the journals was 2.1 (IQR: 1.7–2.9).

**FIGURE 2:**
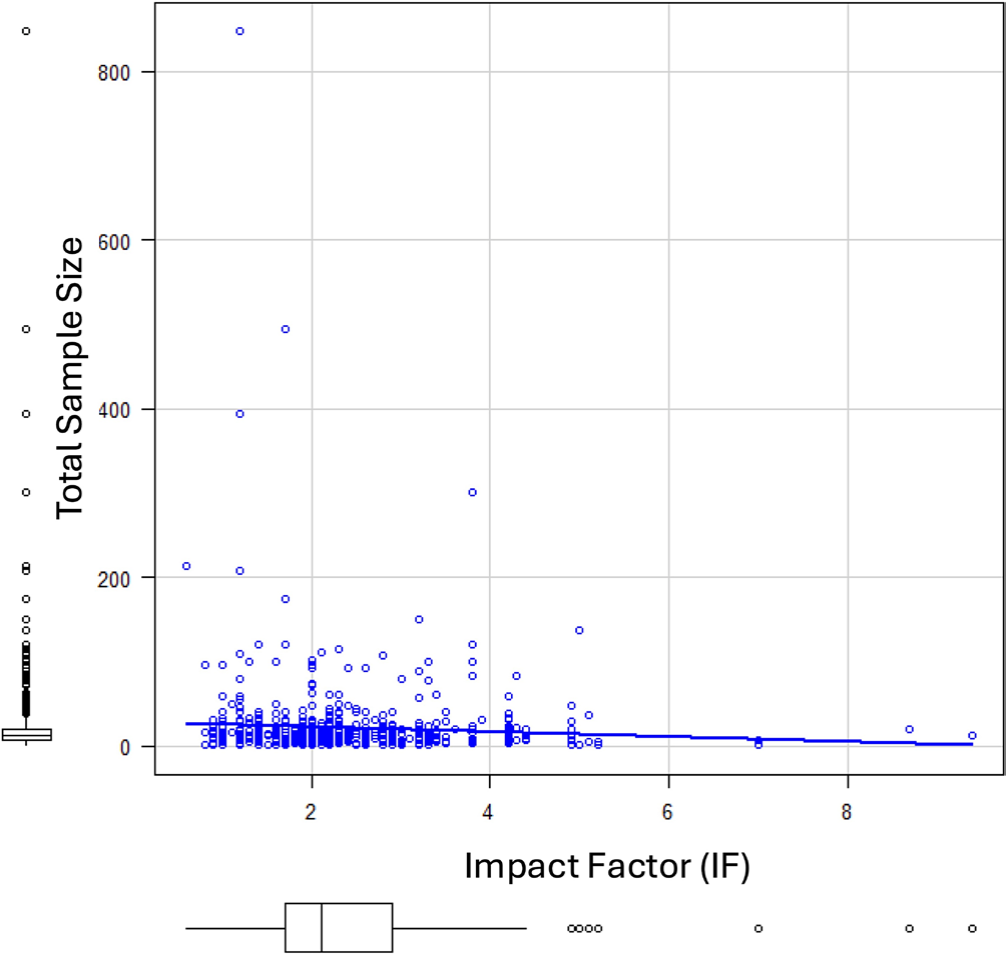
Correlation between sample size and journal impact factor.

No significant correlation was found between the IF and either the sample size or the number of groups (Correlation between IF and sample size: *r* = −0.062, *p* = 0.115; Correlation between IF and number of groups: *r* = −0.037, *p* = 0.648, Figure 2).

## 4 Discussion

In this study, we systematically reviewed 770 cadaveric studies published in 2023–2024 and found that the median sample size was 11.5 (IQR: 7–20), with 47.9% using 10 or fewer samples and 6.4% reporting single‐case studies. These findings provide a valuable benchmark for researchers designing future anatomical studies.

To organize our discussion, we present three key considerations:

### 4.1 What Is the Appropriate Number of Cadaveric Samples for an Anatomical Study?

The debate over sample size in cadaveric research remains unresolved. Although various general research guidelines suggest numbers for pilot or exploratory studies (e.g., 12 samples per group by Julious 2005; 10–40 samples by Hertzog 2008), these are not tailored to the constraints of the anatomical sciences. Our data show that nearly half of the anatomical studies proceed with 10 or fewer samples; however, such numbers may generally be insufficient when classification or subgrouping is applied. As we did not distinguish between case reports and original studies, both were included in the analysis, which may have influenced the results. However, the distinction is unclear—what differentiates a case series of 10 specimens from a qualitative cadaveric study with 10 samples? Given the lack of a standardized definition separating “case report” from “original study,” we deferred this classification issue to future investigations. Notably, 21 articles (2.7%) included more than 100 samples. In most cases, such studies likely required data collection over multiple academic years, reflecting the limited availability of cadaveric specimens in many countries and regions (Iwanaga, Takeda, et al. 2025).

Our linear regression model revealed that studies with 2–6 groups typically failed to meet the recommended total sample size based on statistical estimation, except for limited studies (Shujaat et al. 2023; Iwanaga et al. 2024; Kodera et al. 2024; Manjatika et al. 2023). This suggests a disconnect between the study design and practical feasibility. Nevertheless, by nature, cadaveric research operates under limitations, such as donor scarcity and ethical or cultural factors (Iwanaga, Kim, et al. 2025), which necessitate a more flexible standard. Still, researchers conducting anatomical studies should make efforts to address this gap by increasing the number of samples whenever possible.

### 4.2 What Influences Cadaveric Sample Size?

Unlike animal or clinical studies, the availability of human cadavers varies drastically across countries and institutions. The cultural perceptions of death, legal frameworks, and dissection logistics impact access to specimens. Some studies, such as Behnia et al. (2000), using 669 lingual nerves, reached high sample numbers due to specific legal requirements for autopsy. Others, like Ricci et al. (2024), demonstrated proof‐of‐concept findings using only three specimens.

Therefore, cadaveric sample size is determined by a combination of research purpose (qualitative vs. quantitative), the anatomical structure under investigation, institutional resources, and regional ethics or laws. These constraints must be acknowledged when interpreting the quality or generalizability of anatomical data.

### 4.3 Does Sample Size Correlate With Journal IF?

Interestingly, our correlation analysis revealed no significant relationship between sample size and journal IF. This indicates that publication success in higher‐IF journals is not driven by large sample size but likely by the relevance, novelty, and quality of the findings. Anatomical researchers should be reassured that rigorous methodology and meaningful contributions, rather than large sample size alone, remain central to publication success.

## 5 Conclusion

This review of 770 cadaveric studies provides evidence‐based insights into current trends in anatomical sample size:

- The median sample size was 11.5, and nearly half of the studies used ≤ 10 specimens.
- Studies employing classification into multiple groups often failed to meet the statistically recommended sample size.
- No correlation was found between sample size and journal IF, highlighting the importance of methodological rigor over quantity.

We propose the following minimum standards:

- A total sample size of at least 10 for basic cadaveric studies.
- For studies involving group comparisons, ≥ 5 samples per group, and adherence to regression‐based sample size guidance.

These benchmarks are not intended as rigid requirements, but as references to help guide better‐aligned anatomical study designs.

## Data Availability

Research data are not shared.
